# Surgical Outcomes and Patient Expectations and Satisfaction in Spine Surgery Stratified by Surgeon Age

**DOI:** 10.1001/jamanetworkopen.2025.5984

**Published:** 2025-04-21

**Authors:** Brett Ells, Mayilee Canizares, Raphaële Charest-Morin, Andrew Nataraj, Chris Bailey, Eugene Wai, Alex Soroceanu, Travis Marion, Marcel Dvorak, Y. Raja Rampersaud, Charles Fisher, Zhi Wang, Naj Attabib, Sean Christie, Nicholas Dea, Adrienne Kelly, Supriya Singh, Bernard Larue, Michael Weber, Chris Small, Hamilton Hall, R. Andrew Glennie

**Affiliations:** 1 Dalhousie University, Halifax, Nova Scotia, Canada; 2 University Health Network Toronto, Toronto, Ontario, Canada; 3 University of British Columbia, Vancouver, British Columbia, Canada; 4 University of Alberta, Edmonton, Alberta, Canada; 5 Western University, London, Ontario, Canada; 6 University of Ottawa, Ottawa, Ontario, Canada; 7 University of Calgary, Calgary, Alberta, Canada; 8 Universite de Montreal, Montreal, Quebec, Canada; 9 Sault Area Hospital, Sault Ste Marie, Ontario, Canada; 10 University de Sherbrooke, Sherbrooke, Quebec, Canada; 11 Horizon Health New Brunswick, New Brunswick, Canada; 12 McGill University, Montreal, Quebec, Canada

## Abstract

**Importance:**

There is a paucity of data comparing patient-reported outcomes across surgeon age. Prior work has focused on adverse event rates for surgeon age across a variety of surgical procedures.

**Objective:**

To compare patient-reported outcomes, expectation fulfillment, and satisfaction measures after spine surgery across surgeon age categories.

**Design, Setting, and Participants:**

This retrospective cohort study was conducted at multicentered tertiary referral centers across Canada. Patients with degenerative conditions of the spine were enrolled in a national research network from January 2015 to August 2020. Patients were linked to a demographic survey distributed to spine surgeons who enrolled the patients. Elective surgery for degenerative spine conditions were followed up for a minimum of 1 year after operation. The data were analyzed in January 2024.

**Exposure:**

Surgeons were classified according to their age: younger (age 35-44 years), middle age (45-59 years), and older (≥60 years).

**Main Outcome and Measures:**

The primary outcomes were the Ostwestry Disability Index (ODI) and Neck Disability Index (NDI), numerical pain scores, expectation fulfillment, and overall satisfaction with spine surgery. Baseline demographic and clinical data and surgical procedure complexity were collected. Multivariate logistic regression models were employed, using generalized estimating equations to account for clustering within surgeons, to compare patient outcomes, expectation fulfillment, and satisfaction by surgeon age.

**Results:**

A total of 3421 patients (1236 [36.1%] aged 65 years or older; 1603 female [46.9%]) were included in the study for analysis, with 811 (23.7%) treated by younger surgeons, 1643 (48.0%) by middle-age surgeons, and 967 (28.3%) by older surgeons. There were 2857 procedures of the lumbar spine (83.5%). After accounting for patient demographic, clinical, surgical, and surgeon characteristics, there were no significant differences in disability and pain (ODI and NDI or pain score) at 12 months among younger (mean ODI and NDI score, 25.6; 95% CI, 24.3-26.9; mean pain score, 3.4; 95% CI, 3.2-3.6), middle-age (mean ODI and NDI score, 25.8; 95% CI, 24.9-26.8; mean pain score, 3.3; 95% CI, 3.2-3.4), and older (mean ODI and NDI score, 24.6; 95% CI, 23.4-25.8; mean pain score, 3.4; 95% CI, 3.2-3.6) surgeons. Patients treated by younger (adjusted odds ratio [aOR], 1.57; 95% CI, 1.02-2.40) and middle-age (aOR, 1.41; 95% CI, 1.06-1.86) surgeons reported having all their expectations fulfilled compared with older surgeons. Additionally, patients treated by younger surgeons reported higher satisfaction levels (aOR, 1.29; 95% CI, 1.01-1.69) compared with middle-aged and older surgeons.

**Conclusions and Relevance:**

In this retrospective cohort study of patients who underwent elective spine surgery, there was no difference in outcomes by surgeon age at 1 year, but patients treated by younger surgeons reported higher levels of satisfaction and expectation fulfillment. These findings suggest that spine surgeons of all ages are a valuable resource given similar patient outcomes for all groups.

## Key Points

**Question:**

Is surgeon age associated with patient reported outcomes, expectation fulfillment, and satisfaction with spine surgery?

**Findings:**

In this cohort study of 3421 patients, there were no differences in outcomes for pain or disability at 12 months by surgeon age (younger, 35-44 years; middle age, 45-59 years; older, ≥60 years). Patients reported greater postoperative satisfaction with younger surgeons and patient expectations were met if they were treated by middle-aged or younger surgeons.

**Meaning:**

Surgeon age was found to have no association with functional outcomes but there was a slight preference for younger surgeons with respect to subjective patient experience, suggesting that spine surgeons of all ages are a valuable resource given similar patient outcomes for all groups.

## Teaser

This cohort study analyzes patient-reported outcomes, expectation fulfillment, and satisfaction measures after spine surgery across surgeon age categories among patients who underwent elective spine surgery in Canada.

## Introduction

It is unclear if surgeon age is associated with patient outcomes after spine surgery. More surgeons are working later in life, and it is critical to evaluate whether patient outcomes are similar given the effects of aging on physique and cognition. Older surgeons contribute valuable experience and expertise; however, they are subject to age-related changes in vision, movement, and cognition and the stress associated with the evolution of techniques and equipment in their field of practice.^1^ Although it is essential to identify physicians with impaired abilities to perform their duties, this must be balanced with protecting colleagues from discrimination and ageism, especially in an era where the need for surgeons is ever-increasing.^2^

The association of surgeon volume per year with postoperative outcomes has been well-studied, demonstrating that complications decrease as volume increases.^3,4,5,6,7,8,9,10,11,12,13,14^ Older surgeons (ie, late-career) with a developed patient roster, practice, and experience commonly have higher volume and cases per year than younger surgeons.^15^ However, the surgeon’s age in association with surgical outcomes has been studied less, representing a gap in the literature. Studies have examined the association of surgeon age with postoperative outcomes over several years, using adverse events as outcome variables. These studies found that as age increases, there are either decreasing rates of postoperative complications or no change compared with younger surgeons. Further, these studies have analyzed a very heterogeneous collection of surgeries making it impossible to control for procedural complexity.^15,16,17,18^

At the intersection of the increased need for surgeons to deliver health care services, processes to improve the quality of care, and an aging population of physicians, it is important to understand patient-reported outcomes according to surgeon age categories. It is also critical to determine if surgeons of different ages are performing similarly invasive procedures to ensure that outcomes are not due to changing practice patterns (ie, less complex surgery in later career). The objective of this study was to compare patient-reported outcome measures, expectation fulfillment, and satisfaction by surgeon age, adjusting for patient and surgeon characteristics.

## Methods

This retrospective cohort study used data from the Canadian Spine Outcomes and Research Network (CSORN), a registry of the Canadian Spine Society, to track spine surgery outcomes across Canada. This registry prospectively enrolls patients requiring elective surgical treatment for spinal problems from 70 neurosurgical and orthopedic spine surgeons across 22 sites in 8 provinces in Canada. Patients completed a range of questionnaires before (baseline) and 1 year after surgery. Each participating institution obtained ethics board approval, and all participants consented to participate in the registry. Additionally, surgeons reported diagnostic and clinical features, operative information, and wait times. The study followed the Strengthening the Reporting of Observational Studies in Epidemiology (STROBE) reporting guideline.

The study included patients undergoing elective surgery of the cervical and lumbar spine enrolled from January 2015 through August 2020 who were eligible for follow-up (4580 patients) and had surgery for a degenerative diagnosis (4114 patients). The degenerative spine conditions included degenerative disc disease, disc herniation, spondylolisthesis, and spinal stenosis. Patients with all other conditions including traumatic fractures, infections, and primary or metastatic tumor were excluded from the study.

### Outcomes

At baseline and 1-year follow-up, patients reported their level of pain (back/neck pain and arm/leg pain) using a numerical pain rating scale that varied from 0 (no pain) to 10 (unbearable pain). For analysis, we selected the worst back and neck pain and arm and leg pain reported. The modified Oswestry Disability Index (ODI) and the Neck Disability Index (NDI) for patients with thoracolumbar and cervical diagnoses, respectively, were also collected at these time points. The disability score ranges from 0 to 100 (highest disability). We created 2 variables identifying whether patients had clinically important improvements in pain and disability after surgery.^19^ We used a cutoff point of 2.0 for pain, 12.8 for the ODI, and 15.0 for the NDI.^20,21^

Preoperatively, patients reported their expectations regarding the outcome of the surgery in 6 dimensions: leg and arm pain, back and neck pain, independence in everyday activities, sporting activities and recreation, general physical capacity at work and home, and mental well-being. At 1-year follow-up, for each preoperative expectation domain, they indicated if their preoperative expectations were fulfilled: completely (2), somewhat (1), and not at all (0). We combined responses to this question to create an overall expectation fulfilment variable as (1) all expectations were met at least somewhat, (2) some expectations were met at least somewhat, and (3) none were met. At 1 year following surgery, patients reported their satisfaction with the results of their surgery (extremely satisfied, somewhat satisfied, neither satisfied nor dissatisfied, somewhat dissatisfied, and extremely dissatisfied).

### Main Exposure

We grouped surgeons based on their age. Groups included surgeons aged 35 to 44 years (young), 45 to 59 years (middle age), and 60 years or older (old).

### Covariables

We included several patient and surgeon variables that were hypothesized to be associated with patient-related outcomes. Patient variables included were age, sex, educational level (less than high school, high school, and college or university), labor force status (currently working, in the labor force but not currently working, or not in the labor force), participation in physical activity (active vs inactive), smoking status (current smokers vs not current smokers), body mass index (calculated as weight in kilograms divided by height in meters squared; <25.0, 25.0-29.9, and ≥30.0), number of comorbidities (none, 1-2, and ≥3), principal pathology (stenosis, spondylolisthesis, disc herniation, degenerative disc disorder, and deformity), the type of surgery (fusion vs other), and spine location (thoracolumbar vs cervical). The spine surgical invasiveness index (SSII) is a previously validated instrument which quantifies surgical invasiveness by assigning points per vertebral level of decompression, arthrodesis, and instrumentation from an anterior and/or posterior approach, using a score range from 0 to 48 points.^22,23^ The higher the score, the higher the surgical invasiveness. The index is made up of the sum of 6 weighted surgical components: anterior decompression, anterior fusion, anterior instrumentation, posterior decompression, posterior fusion, and posterior instrumentation, and the weights for each component represent the number of vertebral levels at which each is performed.^22,23,24^ For example, an L4 to L5 posterolateral arthrodesis with L4 and L5 laminectomy and structural graft or cages in the L5 disc space would be scored as anterior decompression = 0, anterior fusion = 2, anterior instrumentation = 2, posterior decompression = 2, and posterior instrumentation = 2, for a total score of 10. For our study, we categorized the SSII score into 3 groups (1-4, 5-9, and ≥10).

A questionnaire was distributed to all spine surgeons who report patient data to the registry (eMethods in Supplement 1). The survey captured surgeon demographics and elements of practice that are not captured within the Canadian Spine Society registry. Surgeon variables included were sex, specialty (orthopedic surgeon or neurosurgeon), years of experience, case volume per year (<200 vs ≥200 surgeries per year), frequency of resident or fellow participation in cases, and percentage of practice dedicated to the spine.

### Statistical Analysis

We report patient demographic and clinical characteristics by surgeon age categories using means and proportions, as appropriate. We used a sequential modeling strategy to assess the association of surgeon age categories with the outcome of interest. We first fitted the unadjusted models with surgeon age categories. In the second step we adjusted for patient and surgeon variables to control for potential confounding. We fitted 2 multivariable logistic regression models for achieving clinically important improvements in disability and pain, respectively; nominal multivariate logistic regression models for fulfillment of expectations (none were met was the reference category); and multivariate ordinal logistic regression models for level of satisfaction (lower to higher). All models included patient and surgeon variables. We fit logistic regression models adjusting for correlations between patients within surgeons by means of the genmod procedure in SAS version 9.4 (SAS Institute), which uses maximum likelihood methods to obtain the model estimates.^25^

We accounted for missing data via maximum likelihood (ML) estimation. ML does not rely on imputing data; instead, it uses each case’s available data to compute the estimates. The ML estimate of a parameter is the value of the parameter that is most likely to have resulted in the observed data. We explored whether the association of surgeons’ age varied by patients’ characteristics by including interactions between these variables. We found no indication of a differential association of surgeon’s age by patient characteristics; therefore, we present models without interactions. Results are presented as odds ratios (ORs) with 95% CIs. All analyses were performed using SAS/STAT software version 9.4 (SAS Institute). The data was definitively analyzed in January 2024. The threshold for statistical significance was considered a 2-sided *P* < .05.

## Results

A total of 693 patients were excluded, including 505 who were lost to follow-up and 188 patients who did not answer questions about satisfaction and fulfillment of expectations. This resulted in a sample of 3421 patients for analysis. There were no significant differences in sociodemographic and health-related factors between the analytical sample and those excluded from the study.

Of 80 surgeons, 63 (78.8%) responded to the questionnaire; surgeons who recruited fewer than 10 patients were excluded. Patients were grouped by surgeon age, with 811 patients (23.7%) in the younger surgeon group, 1643 (48.0%) in the middle-aged surgeon group, and 967 (28.3%) in the older surgeon age group. Of all patients, 1603 (46.9%) were female, 1236 (36.1%) were 65 years or older, 1384 (40.5%) completed at least high school, 567 (16.6%) were current smokers, 1201 (35.1%) had obesity (ie, BMI ≥30), 1740 (50.9%) had 3 or more comorbidities, and 2857 (83.5%) underwent procedures of the lumbar spine (Table 1).

**Table 1.** Sample Descriptive Characteristics by Surgeons’ Age

Patients’ characteristics	Patients stratified by surgeons’ age, No. (%)				*P* value
	Total (N = 3421)	<45 y (n = 811)	45-59 y (n = 1643)	≥60 y	
Age group, y					
<45	689 (20.1)	140 (17.3)	300 (18.3)	249 (25.7)	<.001
45-64	1496 (43.7)	377 (46.5)	709 (43.2)	410 (42.4)	
≥65	1236 (36.1)	294 (36.3)	634 (38.6)	308 (31.9)	
Sex					
Female	1603 (46.9)	393 (48.5)	762 (46.4)	448 (46.3)	.58
Male	1818 (53.1)	418 (51.5)	881 (53.6)	519 (53.7)	
Education level					
<High school	2037 (59.5)	475 (58.6)	970 (59.0)	592 (61.2)	.44
>High school	1384 (40.5)	336 (41.4)	673 (41.0)	375 (38.8)	
Labor force status					
Currently working	1105 (32.3)	228 (28.1)	525 (32.0)	352 (36.4)	.01
Not working	553 (16.2)	134 (16.5)	259 (15.8)	160 (16.5)	
Not in labor force	1733 (50.7)	442 (54.5)	844 (51.4)	447 (46.2)	
Smoking					
Current	567 (16.6)	139 (17.1)	278 (16.9)	150 (15.5)	.56
Non currently smoking	2813 (82.2)	666 (82.1)	1340 (81.6)	807 (83.5)	
Body mass index groups^a^					
Underweight or normal (<25.0)	822 (24.0)	180 (22.2)	409 (24.9)	233 (24.1)	.07
Overweight (25.0-29.9)	1318 (38.5)	295 (36.4)	638 (38.8)	385 (39.8)	
Obesity (≥30.0)	1201 (35.1)	320 (39.5)	551 (33.5)	330 (34.1)	
No. of health problems					
None	272 (8.0)	68 (8.4)	123 (7.5)	81 (8.4)	.28
1-2	1409 (41.2)	311 (38.3)	683 (41.6)	415 (42.9)	
≥3	1740 (50.9)	432 (53.3)	837 (50.9)	471 (48.7)	
Spine location					
Lumbar	2857 (83.5)	684 (84.3)	1342 (81.7)	831 (85.9)	.01
Cervical	564 (16.5)	127 (15.7)	301 (18.3)	136 (14.1)	
Spine surgical invasiveness index score					
1-4	1728 (50.5)	413 (50.9)	858 (52.2)	457 (47.3)	.02
5-9	837 (24.5)	206 (25.4)	400 (24.4)	231 (23.9)	
≥10	856 (25.0)	192 (23.7)	385 (23.4)	279 (28.9)	
Surgery type					
Fusion	1792 (52.4)	419 (51.7)	815 (49.6)	558 (57.7)	.003
Other	1629 (47.6)	392 (48.3)	828 (50.4)	409 (42.3)	
Principal pathology					
Cervical stenosis	313 (9.2)	58 (7.2)	182 (11.1)	73 (7.6)	<.001
Cervical disc herniation	147 (4.3)	35 (4.3)	75 (4.6)	37 (3.8)	
Lumbar stenosis	1099 (32.1)	272 (33.5)	562 (34.3)	265 (27.4)	
Lumbar disc herniation	622 (19.2)	166 (20.5)	299 (18.2)	190 (19.7)	
Spondylolisthesis	881 (25.8)	238 (29.4)	407 (24.8)	236 (24.4)	
Degenerative disc disease	324 (9.5)	42 (5.2)	116 (7.1)	166 (17.2)	
Practice location					
East Canada	666 (19.5)	67 (8.3)	391 (23.8)	208 (21.5)	<.001
Ontario or Quebec	648 (18.9)	226 (27.9)	336 (20.5)	86 (8.9)	
Alberta or Manitoba	1686 (49.3)	381 (47.0)	703 (42.8)	602 (62.3)	
British Columbia	421 (12.3)	137 (16.9)	213 (13.0)	71 (7.3)	

^a^ Body mass index was calculated weight in kilograms divided by height in meters squared.

Older surgeons saw significantly more patients younger than 45 years compared with younger surgeons (249 patients [25.7%] vs 140 patients [17.3%]; *P* < .001). Additionally, older surgeons saw significantly more patients who were currently working compared with younger surgeons (352 patients [36.4%] vs 228 patients [28.1%]), while younger surgeons saw more patients who were not in the labor force than older surgeons (442 patients [54.5%] vs 447 patients [46.2%]) (*P* = .01). In terms of spine location, older surgeons performed more lumbar spine surgeries compared with middle-age surgeons (831 patients [85.9%] vs 1342 patients [81.7%]; *P* = .01). Older surgeons performed more cases of high invasiveness (SSII ≥10) compared with middle-age surgeons (279 patients [28.9%] vs 385 patients [23.4%]; *P* = .02). Additionally, older surgeons performed a significantly higher number of fusions than middle-age surgeons (558 patients [57.7%] vs 815 patients [49.6%]; *P* = .003). Finally, older surgeons saw significantly more patients with degenerative disc disease compared with younger surgeons (166 patients [17.2%] vs 42 patients [5.2%]; *P* < .001) (Table 1).

On average, patients reported improvements in ODI scores and pain at the 1-year mark after their operation. After accounting for patient demographic, clinical, surgical, and surgeon characteristics, there were no significant differences in disability and pain at 12 months among younger (mean ODI and NDI score, 25.6; 95% CI, 24.3-26.9; mean pain score, 3.4; 95% CI, 3.2-3.6), middle-age (mean ODI and NDI score, 25.8; 95% CI, 24.9-26.8; mean pain score, 3.3; 95% CI, 3.2-3.4), and older (mean ODI and NDI score, 24.6; 95% CI, 23.4-25.8; mean pain score, 3.4; 95% CI, 3.2-3.6) surgeons. Additionally, 58.7% of patients (95% CI, 57.0%-60.3%) were extremely satisfied and 27.2% (95% CI, 25.7%-28.7%) were somewhat satisfied with their surgical outcome. Regarding satisfaction with surgery, 28.9% (95% CI, 27.4%-30.5%) reported all their expectations being met and 51.1% (95% CI, 49.4%-52.8%) reported some of their expectations being met. Further information stratified by surgeon age categories can be found in Table 2.

**Table 2.** Outcomes by Surgeon’s Age Categories

Outcome	Patients by surgeon’s age, mean % (95% CI)				*P* value
	All	<45 y	45-59 y	≥60 y	
Pain and disability scores, mean (95% CI)					
Baseline ODI and NDI score	45.5 (45.0-46.1)	46.8 (45.7-47.9)	45.7 (44.9-46.4)	44.3 (43.4-45.3)	.34
12-mo ODI and NDI score	25.4 (24.8-26.1)	25.6 (24.3-26.9)	25.8 (24.9-26.8)	24.6 (23.4-25.8)	.71
Baseline pain score	7.6 (7.6-7.7)	7.6 (7.5-7.7)	7.7 (7.6-7.7)	7.6 (7.5-7.7)	.95
12-mo Pain	3.4 (3.3-3.4)	3.4 (3.2-3.6)	3.3 (3.2-3.4)	3.4 (3.2-3.6)	.69
Expectation fulfillment					
All met	28.9 (27.4-30.5)	28.1 (24.9-31.2)	29.0 (26.8-31.2)	29.5 (26.6-32.4)	.46
Some met but not all	51.1 (49.4-52.8)	53.4 (49.9-56.9)	51.1 (48.6-53.5)	49.1 (45.9-52.3)	
None met	20.0 (18.6-21.4)	18.6 (15.8-21.3)	19.9 (18.0-21.9)	21.4 (18.8-24.0)	
Satisfaction with surgery					
Extremely satisfied	58.7 (57.0-60.3)	62.3 (58.9-65.6)	58.3 (55.9-60.7)	56.4 (53.2-59.5)	.43
Somewhat satisfied	27.2 (25.7-28.7)	25.6 (22.5-28.6)	27.2 (25.0-29.3)	28.8 (25.9-31.6)	
Neither	5.4 (4.6-6.1)	4.0 (2.6-5.4)	5.7 (4.6-6.8)	5.9 (4.4-7.4)	
Somewhat dissatisfied	6.0 (5.2-6.8)	5.5 (3.9-7.1)	5.7 (4.6-6.8)	6.8 (5.2-8.4)	
Extremely dissatisfied	2.8 (2.2-3.3)	2.6 (1.5-3.7)	3.2 (2.3-4.0)	2.2 (1.3-3.1)	

Abbreviations: NDI, Neck Disability Index; ODI, Oswestry Disability Index.

Results from the multivariate logistic regression models analyzing the association of surgeon age with outcomes of interest are summarized in Table 3 with full models presented in eTables 1 to 3 in Supplement 1. The results from models for patient pain and disability improvement did not indicate statistically significant differences in pain and disability improvement after accounting for patient and surgeon characteristics. However, when analyzing the association of surgeon age with patient expectation fulfillment, nominal multivariate logistic regression revealed that patients treated by younger (adjusted OR [aOR], 1.57; 95% CI, 1.02-2.40) and middle-age (aOR, 1.41; 95% CI, 1.06-1.86) surgeons were more likely to have all their expectations fulfilled compared with older surgeons. Additionally, patients treated by younger surgeons were more likely to report higher satisfaction levels (aOR, 1.29; 95% CI, 1.01-1.69) compared with older and middle-age surgeons. Full models for ODI and pain are reported in eTable 1 in Supplement 1. Full models for expectations and satisfaction are reported in eTable 2 and eTable 3 in Supplement 1. A model for surgeon years of experience (ie, <10 years, 11-20 years, and ≥21 years) was also completed and showed similar results as for surgeon age.

**Table 3.** Surgeons’ Age and Patient Outcomes

Outcome	Surgeon age, adjusted OR (95% CI)			*P* value
	≥60 y	35-44 y	45-59 y	
Expectation fulfillment				
None met	1 [Reference]	1 [Reference]	1 [Reference]	.049
All met	1 [Reference]	1.57 (1.02-2.40)	1.41 (1.06-1.86)	
Some met but not all	1 [Reference]	1.48 (0.99-2.20)	1.02 (0.84-1.23)	
Pain and disability				
ODI/NDI (met MCID vs no)	1 [Reference]	1.01 (0.82-1.24)	1.05 (0.87-1.27)	.78
Pain (met MCID vs no)	1 [Reference]	0.98 (0.71-1.36)	1.13 (0.86-1.48)	.35
Satisfaction with surgery (higher satisfaction)	1 [Reference]	1.29 (1.01-1.69)	0.94 (0.77-1.14)	.03

Abbreviations: MCID, minimal clinically important difference; NDI, Neck Disability Index; ODI, Oswestry Disability Index; OR, odds ratio.

## Discussion

This cohort study aimed to better understand the association of spine surgeon age with patient-reported outcome measures after spine surgery, patient expectations, and patient satisfaction. Older surgeons tended to perform more lumbar operations, perform fusion surgery more commonly, operate on younger patients, and operate on a higher rate of patients with degenerative disc disease compared with their younger counterparts. Older surgeons were more likely to have patients with higher SSII scores. Regarding patient-reported outcomes, results demonstrated no difference between surgeon age and patient-reported pain or ODI and NDI scores. Finally, results revealed statistically significant associations of surgeon age with patient expectations and satisfaction. Specifically, patients under the care of younger surgeons reported more of their expectations being met and higher satisfaction rates than those under the care of older surgeons.

Prior reports of patient outcomes based on physician age demonstrate conflicting results. A large administrative database review by Tsugawa et al^18^ demonstrated a higher 30-day mortality rate for patients treated by older hospitalist physicians. Satkunasivam et al^15^ specifically evaluated surgeon outcomes and demonstrated a linear improvement in postoperative death, readmission, and complications with increasing surgeon age based on 25 common surgical procedures in a large provincial database. A major advantage of the current work is that the CSORN registry has data fields specifically allowing to account and calculate the intensity or invasiveness of the procedure. Other administrative database studies can only control for surgeon volumes, and although there may be patient satisfaction scores, very few use formal validated patient reported outcome measures.

Over the past 2 decades, there has been increased attention to communication skill development and training during medical school.^26^ Younger physicians may have acquired communication skills that prove useful when setting realistic expectations with patients before deciding to proceed with surgery; this may leave patients feeling more satisfied postsurgery, as opposed to patients who needed to have expectations more clearly communicated before undergoing spine surgery. Alternatively, patients may perceive younger surgeons as being up to date with respect to modern techniques and innovation; this may bias patients to reporting greater degrees of satisfaction if they perceive their care as cutting edge.

An interesting finding in this study was that older surgeons operated on a higher proportion of patients with increased SSII scores. It has been demonstrated that with an increase rate of SSII, there is a high probability for adverse outcomes such as infection and blood loss.^22,24^ Patients with an elevated SSII index are more complicated surgical cases and are possibly more complex patients to begin with. In addition to increased SSII, we found that older surgeons operated on a higher proportion of patients with degenerative disc disease. It has been found that while surgical intervention is beneficial for radicular pain, it is less consistent in relieving nonradiating lower back pain.^27,28^ The SSII and degenerative disc disease did not affect expectation fulfillment nor satisfaction overall within the full model; however and therefore, these factors are less likely explanatory for differences in expectation fulfillment and satisfaction based on age category.

### Strengths and Limitations

Our findings make an important contribution to the literature because we examined the association of surgeon age with pain and disability outcomes, expectation fulfillment, and patient satisfaction with surgery, an area that is understudied. The strengths of this study are that the data used come from a national registry with a large sample size, longitudinal design, and a high follow-up rate. We included a wide range of diagnoses and surgical techniques and invasiveness that are generalizable to the broad surgical spine population. The study included standardized tools (ODI and NDI) to measure patient outcomes that are both reliable and of high validity. The patient-reported outcome measures included in this study were examined over a medium-term follow-up (eg, 1 year),^29^ which allowed for the proper assessment of maximal levels of patient improvement in pain and disability.

A substantial limitation with the current study is surgeon age differential at the time of questionnaire completion vs how old they were when the patient had surgery. A surgeon may have been in one of the younger age categories if the patient was treated earlier in the study period. For example, if the surgeon was 58 years in 2015 but completed the questionnaire in 2022, then they would have been included in the older cohort. Although this does not likely impact the patient reported outcome finding, it may impact the differences that were observed in satisfaction and expectation fulfillment scores. The same effect could be present on the other younger side, where surgeons who were 40 years at the time of the questionnaire were actually younger at the time of the surgery they performed. Additionally, we used chronological age rather than years of experience, which may have an impact on how our results are interpreted. Although surgeon experience is accounted for within our analysis, there are surgeons who enter medicine later in life; this makes it less likely that any difference in satisfaction or expectation fulfillment were due to any differences in training despite the close correlation with surgeon age and experience within the group.

The possibility of other unmeasured confounders may influence reporting of fulfillment of expectations and patient satisfaction as well. Although other health problems are included within the analysis, these are not specific. For example, patients who have congestive heart failure vs major depression would both have the same classification of health problem. Also, patient satisfaction is likely related to more than the surgery (eg, anesthesia, the cleanliness of the hospital, and the nursing staff both in and out of hospital). These confounders were not accounted for when considering patient satisfaction.

Another limitation is that the sample of patients for this study was extracted from the CSORN database, which includes both academic and community centers. In other words, the sample of patients was rather heterogenous. There was also a disproportionate part of the sample from sites in Alberta and Manitoba, where sites may have had better provincial resources for patient capture; this seems to have reflected in more thorough enrollment within the registry and may reflect the local surgeons’ motivation for enrolling in the registry. Ontario is the most populous province in Canada. Given its lower contribution to the overall number of patients for this study, it may suggest that the sample is more reflective of sites that can fund the rigorous patient follow-up within the registry and not the overall population.

## Conclusions

In this retrospective cohort study of patients who underwent elective spine surgery, those treated by younger and middle-age surgeons had greater odds of having their expectations met. Patients treated by younger surgeons had greater odds of being more satisfied. There was no difference in patient-reported outcomes of pain and disability between older spine surgeons and their younger counterparts. This study reinforces that all spine surgeons remain an impactful and, thus, valuable resource to our health care systems where they did not have inferior outcomes compared with their younger counterparts in keeping with the literature. Future research should focus on qualitative patient outcomes to further determine why younger surgeons’ satisfaction rates are slightly higher.

## Notes

Supplement 1. **eMethods.** Surgeon Age Questionnaire **eTable 1.** Full Model for ODI and Pain **eTable 2.** Full Model for Expectations Met **eTable 3.** Full Model for Satisfaction With Surgery

Supplement 2.
Data Sharing Statement
