# Supplementary material for: Surgical Outcomes and Patient Expectations and Satisfaction in Spine Surgery Stratified by Surgeon Age
Source: JAMA Netw Open. 2025 Apr 21;8(4):e255984. doi: 10.1001/jamanetworkopen.2025.5984 (PMC12013352; doi:10.1001/jamanetworkopen.2025.5984)
Supplement: Supplement 1. — eMethods. Surgeon Age Questionnaire eTable 1. Full Model for ODI and Pain eTable 2. Full Model for Expectations Met eTable 3. Full Model for Satisfaction With Surgery [file jamanetwopen-e255984-s001.pdf]

Supplemental Online Content

Ells B, Canizares M, Charest-Morin R, et al. Surgical outcomes, patient expectations and satisfaction in spine surgery stratiied by surgeon age. *JAMA Netw. Open*. 2025;8(4):e255984. doi:10.1001/jamanetworkopen.2025.5984

eMethods. Surgeon Age Questionnaire

eTable 1. Full Model for ODI and Pain

eTable 2. Full Model for Expectations Met

eTable 3. Full Model for Satisfaction With Surgery

This supplemental material has been provided by the authors to give readers additional information about their work.

© 2025 Ells B et al. *JAMA Network Open*.

eMethods. Surgeon Age Questionnaire

1. What is your age?

2. What is your sex?

    a. Male
    b. Female
    c. Other
    d. Prefer not to say

3. How many years have you been in independent practice?

4. How many spine surgeries do you complete per year?

    a. <200
    b. >201

5. What is your surgical specialty?

    a. Orthopedic surgery
    b. Neurosurgery

6. What percentage of your practice is dedicated to spine surgery alone?

    a. 0-25%
    b. 26-50%
    c. 51-75%
    d. 76-100%

© 2025 Ells B et al. *JAMA Network Open*.

7. What percentage of spine surgeries you perform involve fellows?
    a. 0-25%
    b. 26-50%
    c. 51-75%
    d. 76-100%

8. What percentage of spine surgeries you perform involve residents?

    a. 0-25%
    b. 26-50%
    c. 51-75%
    d. 76-100%

© 2025 Ells B et al. *JAMA Network Open*.

eTable 1. Full Model for ODI and Pain

	ODI (Achieved MCID vs. did not)				Pain (Achieved MCID vs. did not)			
	**OR**	**LCL**	**UCL**	**p-value**	**OR**	**LCL**	**UCL**	**p-value**
**Surgeon characteristics**								
Surgeons age (Ref: 60+)								
35-44	1.01	0.82	1.24	0.92	0.98	0.71	1.36	0.91
45-59	1.05	0.87	1.27	0.62	1.13	0.86	1.48	0.38
Specialty: Orthopedic surgeon (Ref: neurosurgeon)	1.06	0.87	1.29	0.55	**1.36**	**1.08**	**1.70**	**0.008**
Surgeon volume: surgeries/year (Ref: <200)	1.08	0.88	1.32	0.48	1.09	0.85	1.39	0.50
**Patient characteristics**								
Patient's age (Ref: <45)								
45-64	**0.76**	**0.63**	**0.93**	**0.008**	**0.64**	**0.47**	**0.86**	**0.0037**
65+	**0.78**	**0.63**	**0.96**	**0.02**	**0.69**	**0.50**	**0.95**	**0.0214**
Sex (Ref: Male)	0.97	0.85	1.11	0.64	1.01	0.84	1.21	0.9189
Education (Ref: <high school)	**1.32**	**1.15**	**1.51**	**<.0001**	**1.13**	**1.01**	**1.27**	**0.0313**
Labour force status (Ref: Not in LBF)								
Currently working	**1.51**	**1.24**	**1.83**	**<.0001**	**1.37**	**1.17**	**1.61**	**<0.001**
In LBF but not working	0.98	0.80	1.19	0.82	0.91	0.72	1.13	0.39
Not reported	2.04	0.62	6.75	0.24	0.62	0.24	1.56	0.3073
Currently smoking (Ref: non-smokers)	**0.75**	**0.64**	**0.89**	**0.001**	**0.73**	**0.59**	**0.90**	**0.003**
BMI (Ref: Normal)								
Overweight	1.04	0.88	1.23	0.61	1.11	0.80	1.54	0.52
Obese	1.09	0.89	1.34	0.41	1.03	0.80	1.34	0.81
# of comorbidities (Ref: None)								
1-2	0.76	0.55	1.05	0.10	1.40	0.97	2.01	0.07
3+	**0.50**	**0.36**	**0.70**	**<.001**	1.22	0.83	1.80	0.31

© 2025 Ells B et al. *JAMA Network Open*.

Fusion (yes vs. no)	1.13	0.85	1.50	0.40	1.20	0.80	1.79	0.38
Principal pathology (Ref: Lumbar Stenosis)								
Cervical stenosis	1.06	0.80	1.40	0.67	**0.69**	**0.54**	**0.88**	**0.003**
Cervical DH	1.74	1.09	2.78	**0.021**	0.93	0.60	1.46	0.76
Lumbar DH	1.57	1.22	2.01	**<0.001**	**1.36**	**1.14**	**1.63**	**<0.001**
Spondylolisthesis	1.67	1.40	2.00	**<.001**	**1.63**	**1.20**	**2.21**	**0.002**
DDD	1.05	0.83	1.31	0.70	0.77	0.50	1.17	0.22
SSII (Ref: 1-4)								
5-9	0.83	0.65	1.07	0.15	0.90	0.63	1.29	0.57
10+	**0.76**	**0.58**	**0.99**	**0.04**	0.98	0.66	1.46	0.92

© 2025 Ells B et al. *JAMA Network Open*.

eTable 2. Full Model for Expectations Met

(Results from Nominal Logistic Regression)

	Expectations met							
	All met vs. None				Some met vs. None			
	OR	LCL	UCL	p-value	OR	LCL	UCL	p-value
**Surgeon characteristics**								
Surgeons age (Ref: 60+)								
35-44	**1.57**	**1.02**	**2.40**	**0.04**	**1.41**	**1.06**	**1.86**	**0.02**
45-59	1.48	0.99	2.20	0.06	1.02	0.84	1.23	0.86
Specialty: Orthopedic surgeon (Ref: neurosurgeon)	1.06	0.68	1.65	0.81	1.14	0.89	1.45	0.30
Surgeon volume: surgeries/year (Ref: <200)	0.97	0.64	1.47	0.89	1.02	0.82	1.28	0.85
**Patient characteristics**								
Patient's age (Ref: <45)								
45-64	1.05	0.78	1.43	0.74	1.04	0.75	1.44	0.82
65+	1.23	0.81	1.88	0.33	1.02	0.69	1.49	0.93
Sex (Ref: Male)	1.19	0.94	1.52	0.15	0.92	0.74	1.15	0.46
Education (Ref: <high school)	**1.38**	**1.08**	**1.77**	**<0.01**	**1.34**	**1.14**	**1.57**	**0.0003**
Labour force status (Ref: Not in LBF)								
Currently working	**1.43**	**1.01**	**2.02**	**0.04**	1.23	0.92	1.64	0.16
In LBF but not working	0.74	0.46	1.18	0.21	0.76	0.53	1.07	0.11
Not reported	0.40	0.08	1.96	0.26	0.52	0.18	1.46	0.21
Currently smoking (Ref: non-smokers)	0.73	0.50	1.06	0.10	0.74	0.56	0.99	0.04
BMI (Ref: Normal)								
Overweight	1.16	0.87	1.56	0.31	1.04	0.78	1.40	0.77
Obese	0.92	0.60	1.42	0.71	1.04	0.76	1.42	0.82
# of comorbidities (Ref: None)								
1-2	0.81	0.54	1.21	0.29	0.93	0.61	1.40	0.72

© 2025 Ells B et al. *JAMA Network Open*.

3+	0.54	0.36	0.82	0.004	0.87	0.57	1.34	0.52
Spine locations (Ref: Cervical)	**0.62**	**0.39**	**0.98**	**0.04**	**0.69**	**0.53**	**0.90**	**0.006**
Fusion (yes vs. no)	0.84	0.49	1.44	0.52	0.81	0.58	1.12	0.20
Principal pathology (Ref: Lumbar Stenosis)								
Cervical stenosis	1.17	0.58	2.36	0.67	1.39	1.00	1.93	0.05
Cervical DH	**2.89**	**1.23**	**6.81**	**0.02**	**1.92**	**1.09**	**3.37**	**0.02**
Lumbar DH	0.92	0.65	1.30	0.64	0.82	0.65	1.04	0.10
Spondylolisthesis	**1.87**	**1.14**	**3.05**	**0.01**	1.22	0.98	1.53	0.08
DDD	1.18	0.53	2.64	0.68	1.05	0.70	1.58	0.81
SSII (ref:1-4)								
5-9	0.96	0.56	1.63	0.87	1.19	0.84	1.67	0.32
10+	0.61	0.37	1.01	0.05	0.92	0.66	1.30	0.65
ODI (Achieved MCID vs. No)	**12.48**	**9.36**	**16.64**	**<.001**	**2.95**	**2.38**	**3.65**	**<.001**
Pain (Achieved MCID vs. No)	**6.83**	**4.73**	**9.85**	**<.001**	**1.91**	**1.49**	**2.45**	**<.001**

© 2025 Ells B et al. *JAMA Network Open*.

eTable 3. Full Model for Satisfaction With Surgery

(Results from ordinal logistic regression with OR>1 indicating higher levels of satisfaction)

	Satisfaction			
	**OR**	**LCL**	**UCL**	**p-value**
**Surgeon characteristics**				
Surgeons age (Ref: 60+)				
35-44	1.29	1.01	1.69	0.04
45-59	0.94	0.77	1.14	0.51
Specialty: Orthopedic surgeon (Ref: neurosurgeon)	0.99	0.82	1.19	0.92
Surgeon volume: surgeries/year (Ref: <200)	0.94	0.76	1.16	0.56
**Patient characteristics**				
Patient's age (Ref: <45)				
45-64	1.02	0.83	1.25	0.87
65+	1.17	0.92	1.49	0.21
Sex (Ref: Male)	1.13	0.96	1.32	0.13
Education (Ref: <high school)	0.99	0.84	1.16	0.89
Labour force status (Ref: Not in LBF)				
Currently working	1.14	0.92	1.42	0.24
In LBF but not working	1.12	0.84	1.48	0.44
Not reported	1.03	0.48	2.20	0.94
Currently smoking (Ref: non-smokers)	0.94	0.77	1.14	0.52
BMI (Ref: Normal)				
Overweight	1.05	0.84	1.32	0.65
Obese	1.12	0.87	1.44	0.38

© 2025 Ells B et al. *JAMA Network Open*.

# of comorbidities (Ref: None)				
1-2	0.79	0.57	1.10	0.16
3+	0.79	0.59	1.07	0.12
Spine locations (Ref: Cervical)	**0.68**	**0.54**	**0.85**	**<0.001**
Fusion (yes vs. no)	1.06	0.62	1.81	0.84
Principal pathology (Ref: Lumbar Stenosis)				
Cervical stenosis	**1.61**	**1.26**	**2.05**	**<0.001**
Cervical DH	1.44	0.92	2.26	0.11
Lumbar DH	**0.77**	**0.63**	**0.94**	**0.01**
Spondylolisthesis	1.30	0.97	1.74	0.08
DDD	0.98	0.71	1.35	0.92
SSII (ref:1-4)				
5-9	1.10	0.66	1.82	0.72
10+	0.97	0.57	1.65	0.90
Expectations met (Ref: None met)				
All met	**38.60**	**27.23**	**54.73**	**<.001**
Some met	**4.05**	**3.47**	**4.74**	**<.001**
ODI (Achieved MCID vs. No)	**3.45**	**2.88**	**4.13**	**<.001**
Pain (Achieved MCID vs. No)	**2.03**	**1.58**	**2.62**	**<.001**

© 2025 Ells B et al. *JAMA Network Open*.
