# Supplementary material for: Surgical Outcomes and Patient Expectations and Satisfaction in Spine Surgery Stratified by Surgeon Age
Source: JAMA Netw Open. 2025 Apr 21;8(4):e255984. doi: 10.1001/jamanetworkopen.2025.5984 (PMC12013352; doi:10.1001/jamanetworkopen.2025.5984)
Supplement: Supplement 2. — Data Sharing Statement [file jamanetwopen-e255984-s002.pdf]

Data Sharing Statement

Ells. Surgical Outcomes and Patient Expectations and Satisfaction in Spine Surgery Stratified by Surgeon Age. *JAMA Netw Open*. Published April 21, 2025.
doi:10.1001/jamanetworkopen.2025.5984

Data
Data available: No
